# On the mechanisms of epidermal stemness and differentiation

**DOI:** 10.1038/s41540-025-00581-3

**Published:** 2025-09-30

**Authors:** Saumya Shukla, Raghvendra Singh

**Affiliations:** Department of Chemical Engineering, Indian Institute of Technology Kanpur, Kanpur, India

**Keywords:** Developmental biology, Stem cells, Systems biology

## Abstract

High Wnt and low Notch activities characterize epidermal stem cells (SCs), while low Wnt and high Notch activities characterize the terminally differentiated epidermal cells (TDCs). However, the mechanism by which transit amplifying cells (TACs) are induced to become terminally differentiated remains unclear. Our analysis suggests that oscillations in Wnt, Notch, and YAP/TAZ activities lead to the production of TDCs from TACs. Furthermore, the role of stem cell markers in epidermal differentiation, regeneration, and the functional aspects of the epidermis remains unclear. Here, based on the ability of the epidermal SCs to induce the differentiation of TACs, we characterize the SCs as having the expression of Notch ligand, Delta, higher than a critical value. Further, we have functionally defined the critical value of the Delta expression by SCs. Our paper may have general implications for the stemness and differentiation of other tissues.

## Introduction

A network of signaling pathways coordinates the cell fate and behavior. The evolutionarily conserved Notch, Wnt, and YAP pathways control tissue dynamics and homeostasis^1,2^. The Wnt signaling is required for the proliferation and self-renewal of epidermal stem cells (SCs)^3^. Further, Axin2, a Wnt target gene, marks the epidermal SCs. These cells produce both the Wnt signal, acting in an autocrine manner, and the long-range Wnt inhibitors^3^. Furthermore, Axin2-expressing cells constitute the majority of the basal layer of the epidermis^3^. On the other hand, the epidermal SCs divide asymmetrically and differentiate in a Notch-dependent manner^4^. Further, Wnt pathway activation opposes epidermal differentiation^3^. Thus, the Wnt pathway is responsible for maintaining epidermal stemness, while activation of the Notch pathway causes epidermal differentiation.

The integrin-mediated signaling causes the nuclear localization of YAP/TAZ^5^. Similarly, the rigidity of the extracellular matrix (ECM) activates YAP/TAZ^6^. Mechano-activation of YAP/TAZ enhances epidermal stemness by inhibiting Notch signaling^6^. In this context, nuclear YAP/TAZ binds to distant enhancers, causing the expression of Delta-like ligands (DLLs), serving as ‘in *cis*’ inhibitors of Notch^6^. Thus, integrin signaling regulates epidermal stemness by inhibiting the Notch pathway^6^ through nuclear YAP/TAZ. Further, since Wnt activity has been found to maintain stemness, while YAP/TAZ also maintains epidermal stemness by inhibiting the notch pathway and causing expression of DLLs, and the notch activity causes epidermal differentiation, the Wnt, YAP/TAZ, and the Notch pathways are the master regulators of epidermal stemness and differentiation.

A required property of stem cells is their potential to differentiate. Peaks and troughs of oscillations may create dynamic conditions in stem cells, allowing stemness to be maintained and stem cells to proliferate under peak/trough conditions. At the same time, the potential to differentiate is also kept under trough/peak conditions, and proliferation and differentiation characteristics may be important for the maintenance of stem cells. Interestingly, core clock genes in human epidermal stem cells oscillate, establishing distinct temporal intervals during the 24-h day period^7^. Each of these successive clock waves, causing the expression of subsets of genes, establishes different temporal predispositions of epidermal stem cells to respond to cues that cause their proliferation or differentiation^7^. Further, in these cells, genes involved in the Notch and Wnt pathways e.g., Notch3, Notch4, Hes4, Hes6, Wnt9a, Wnt10a, TCF7, FZD3, and FZD10 have been found to oscillate^7^. On the other hand, YAP has been shown to balance growth and differentiation in the skin^8^. Further, YAP activated by integrin signaling has been shown to drive the notch signaling to control the stem cell fate specification: to remain undifferentiated and grow, or to activate a terminal differentiation program^6^. Thus, epidermal SCs and the transit amplifying cells (TACs) having integrin signaling are driven by YAP signaling in the epidermal differentiation process.

Although many genes of the Wnt and Notch pathways oscillate in the basal keratinocytes, their functional relevance in the context of epidermal differentiation lacks clarity. Toward this end, we made a simple mechanistic model of this process based on feedback loops among the key regulatory pathways of epidermal stem cell proliferation and differentiation, namely, the Wnt, Notch, and YAP pathways. Although a more complex model involving multiple target genes of these pathways will provide insight into the molecular changes that occur during the differentiation, our model describes the interplay among the master regulators of this process. Previously, we gave a stochastic model of SCs and TACs in the basal layer^9^. According to that study, four cell types exist in the epidermis’s basal layer. These are LGR6 + /Lrig1 + , LGR6 + /Lrig1-, LGR6-/Lrig1 + , LGR6-/Lrig1-^9^. LGR6+ cells are the SCs^10^. In contrast, LGR6- cells are the TACs. Lrig1+ cells are generally quiescent and may differentiate in the presence of EGF/FGF^11^. The four cell types stochastically interconvert among themself through symmetric and asymmetric cell divisions^9^. The model successfully explained the basal layer’s observed composition, homeostasis, and self-renewal, and supported a four-cell type SC-TAC model of the basal layer^9^. In the present study, we give a model of the production of terminally differentiated cells (TDCs) from TACs based on SC-TAC interaction.

## Results

### A critical amount of expression of the Notch ligand, Delta, is required for epidermal stemness

From Eq. (7) (“Method” section), at the steady state $\left(\frac{{{\rm{dC}}}_{{\rm{TDC}}}}{{\rm{dt}}}=0\right)$,

1 $${{\rm{C}}}_{{\rm{TDC}}}=\frac{{{\rm{k}}}_{1}{\rm{d}}}{{{\rm{k}}}_{{\rm{d}}1}}-{{\rm{k}}}_{2}$$

where, ${{\rm{C}}}_{{\rm{TDC}}}$ is the number density of TDCs, and d is the concentration of the Notch ligands, Delta/DLL, on the stem cell surface. Furthermore, according to Eq. (7) (“Method” section), for a nontrivial epidermis, the epidermis locks onto the relationship given by Eq. (1).

**Fig. 1:**
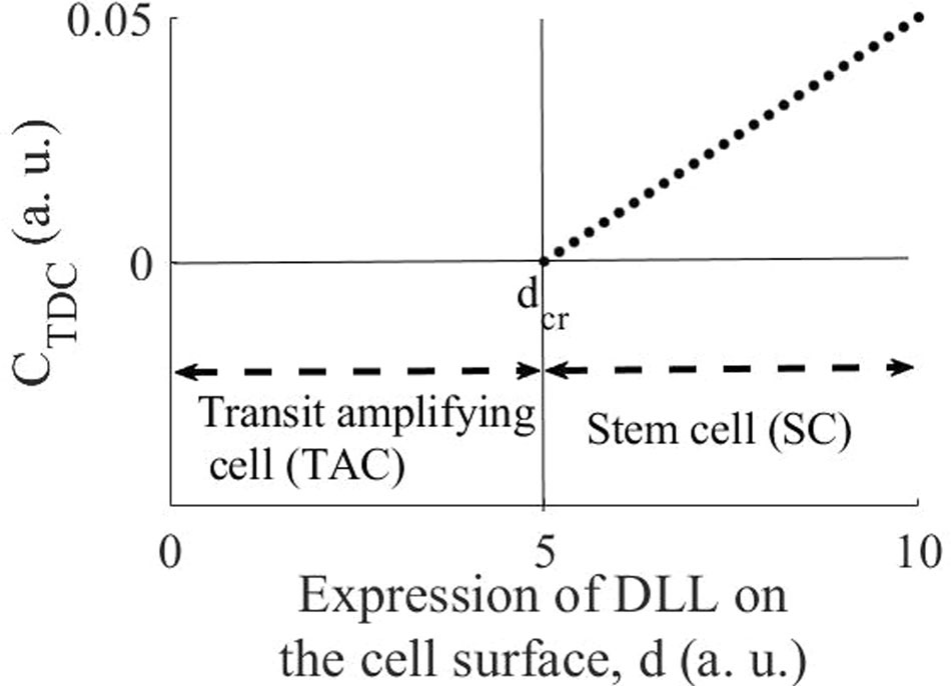
The relationship between the number density of the TDCs and expression of the Notch ligand, DLL, by the epidermal SCs. A linear relationship between the number density of the TDCs (C_TDC_) and expression of the Notch ligand, DLL, has been suggested, consistent with Lowell et. al^12^. Epidermal SCs must express DLL higher than a critical value, d_cr_, so that C_TDC_ > 0. The cells that express DLL lower than d_cr_ are the TACs, in which the ‘in *cis*’ inhibition of the Notch pathway is ineffective due to the lower expression of DLL, so that the Notch pathway can be activated in these cells by the interaction with the epidermal SCs. The plot has been made for $\frac{{{\rm{k}}}_{1}}{{{\rm{k}}}_{{\rm{d}}1}}=0.01$ and ${{\rm{k}}}_{2}=0.05$. a. u.: arbitrary units.

Therefore, at the steady state, the number density of the TDCs increases linearly with the expression of DLL on the epidermal stem cells (Fig. 1). The critical value of DLL, d_Cr_, is when ${C}_{{TDC}}=0$ (Fig. 1), i.e., when the SC cannot produce the TDC from an interacting TAC. Thus, from Eq. (1),

2 $${{\rm{d}}}_{{\rm{Cr}}}=\frac{{{\rm{k}}}_{2}{{\rm{k}}}_{{\rm{d}}1}}{{{\rm{k}}}_{1}}$$

Since an epidermal stem cell is a cell that can induce terminal differentiation in a TAC, for an epidermal stem cell,

3 $$d\ge {d}_{{Cr}}=\frac{{k}_{2}{\,k}_{d1}}{{k}_{1}}$$

or,

4 $${\rm{d}}\ge {{\rm{d}}}_{{\rm{Cr}}}=\frac{\begin{array}{c}{\rm{The\; product\; of\; the\; fold\; decrease\; in\; the\; growth\; rate\; of\; the\; TDCs}}\\ {\rm{due\; to\; Notch\; pathway\; independent\; apoptosis}}\left({{\rm{k}}}_{2}\right){\rm{and\; rate\; of\; movement\; of}}\\ {\rm{the\; the\; TDCs\; to\; the\; suprabasal\; layers}}\left({{\rm{k}}}_{{\rm{d}}1}\right)\end{array}}{\begin{array}{c}{\rm{The\; rate\; of\; generation\; of\; the\; TDCs\; from\; the\; TACs}}\\ {\rm{due\; to\; the\; activation\; of\; Notch\; pathway\; in\; the\; TACs\; by}}\\ {\rm{the\; epidermal\; stem\; cells}}\left({{\rm{k}}}_{1}\right)\end{array}}$$

or,

5 $${\rm{d}}\ge {{\rm{d}}}_{{\rm{Cr}}}=\frac{{\rm{Rate\; of\; loss\; of\; the\; TDCs}}}{{\rm{Rate\; of\; generation\; of\; the\; TDCs}}}$$

The cells that have lower than ${{\rm{d}}}_{{\rm{Cr}}}$ expression of Delta/DLL can be characterized as the TACs (Fig. 1) since in these cells the ‘in *cis*’ inhibition of the Notch receptor will be quite low due to the lower expression of DLL. Thus, in TACs, the Notch activity can be induced, causing their terminal differentiation.

Consistent with our analysis, Lowell et. al have found that all living layers of the epidermis express Notch1 while Delta1 expression was confined to the basal layer^12^. Delta1 expression in epidermal stem cells protects them against the differentiation signal by blocking the Notch pathway, and causes differentiation of cells that reside at the edges of the stem cell clusters^12^.

### Steady states of terminally differentiated cells, C_TDC_

Equation 7 (“Method” section) has two steady states: 1. ${C}_{{TDC}}=0$ and 2. C_TDC,_ given by Eq. 1, i.e.,

$${{\rm{C}}}_{{\rm{TDC}}}=\frac{{{\rm{k}}}_{1}{\rm{d}}}{{{\rm{k}}}_{{\rm{d}}1}}-{{\rm{k}}}_{2}$$

The second steady state is positive (i.e., ${C}_{{TDC}} > 0$)_,_ when $d > {d}_{{Cr}}$.

When the system is at the second steady state, if we perturb the system toward the right of the steady state, the first term, i.e., $\frac{{{\rm{k}}}_{1}{\rm{d}}}{{{\rm{k}}}_{2}+{{\rm{C}}}_{{\rm{TDC}}}}$, in Eq. (7) becomes lower than the second term, i.e., ${{\rm{k}}}_{{\rm{d}}1}$. Then, $\frac{{{\rm{dC}}}_{{\rm{TDC}}}}{{\rm{dt}}}$ becomes negative and C_TDC_ decreases, moving back to the second steady state. Similarly, when the system is at the second steady state, if we perturb the system toward the left of the steady state, the first term, i.e., $\frac{{{\rm{k}}}_{1}{\rm{d}}}{{{\rm{k}}}_{2}+{{\rm{C}}}_{{\rm{TDC}}}}$, in Eq. 7 becomes higher than the second term, i.e., ${{\rm{k}}}_{{\rm{d}}1}$. Then, $\frac{{{\rm{dC}}}_{{\rm{TDC}}}}{{\rm{dt}}}$ becomes positive and C_TDC_ increases, moving back to the second steady state. Thus, the second steady state, ${C}_{{TDC}} > 0$_,_ given by Eq. (1), i.e., ${{\rm{C}}}_{{\rm{TDC}}}=\frac{{{\rm{k}}}_{1}{\rm{d}}}{{{\rm{k}}}_{{\rm{d}}1}}-{{\rm{k}}}_{2}$, is stable while the first steady state, ${C}_{{TDC}}=0$, is unstable.

When d equals d_cr_, the second steady state also becomes zero, i.e., ${C}_{{TDC}}=0$, and the two steady states merge into a single stable steady state ${C}_{{TDC}}=0$.

### Notch, Wnt, and YAP activities in TAC and Wnt activity in the epidermal stem cell oscillate

**Fig. 2:**
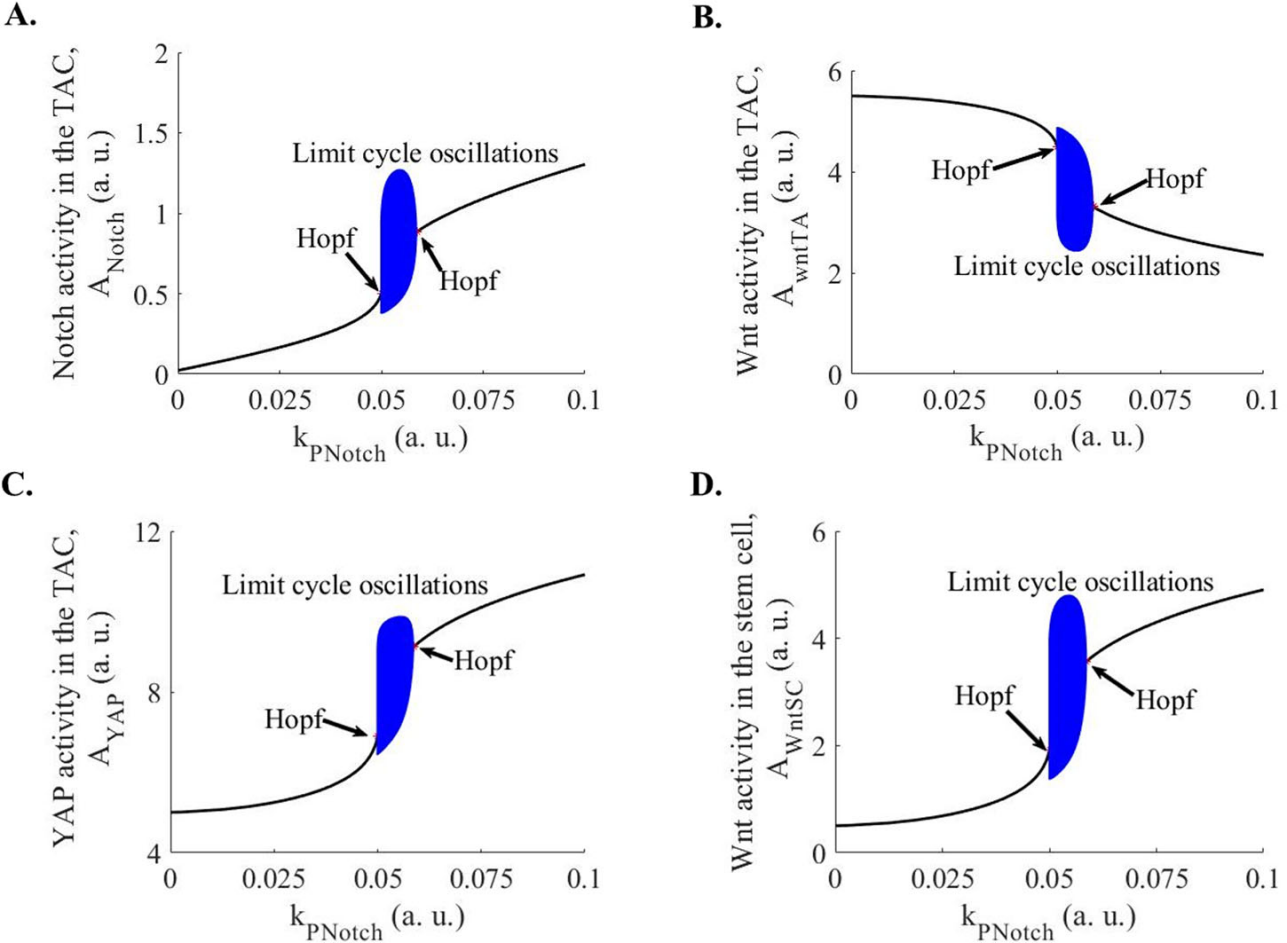
Bifurcation diagrams of the Notch, Wnt, and nuclear YAP/TAZ activities in the TACs, and Wnt activity in the epidermal SCs, which interact with TACs through the Delta-Notch interaction. **A** A bifurcation diagram of the Notch activity in TACs as a function of the generation of the Notch activity, the parameter k_PNotch_, in the TACs has been shown. Between two Hopf bifurcation points, limit cycle oscillations have been observed. **B** A bifurcation diagram of the Wnt activity in TACs as a function of the generation of the Notch activity, the parameter k_PNotch_, in TACs, has been shown. Between two Hopf bifurcation points, limit cycle oscillations have been observed. **C** A bifurcation diagram of the nuclear YAP activity in TACs as a function of generation of the Notch activity, the parameter k_PNotch_, in TACs, has been shown. Between the two Hopf bifurcation points, limit cycle oscillations have been observed. **D** A bifurcation diagram of the Wnt activity in SCs as a function of the generation of the Notch activity, the parameter k_PNotch_, in TACs, has been shown. Between the two Hopf bifurcation points, limit cycle oscillations have been observed. The plots have been made for ${k}_{{Notch}}=1$, ${k}_{{\rm{WntSC}}}=1.4,{k}_{{\rm{WntTA}}}=1,{k}_{{YAP}}=0.188$, ${k}_{{dNotch}}=0.2$, ${k}_{{\rm{dWntSC}}}=0.2$, ${k}_{{\rm{dWntTA}}}=0.2,$ ${k}_{{dYAP}}=0.02$, ${k}_{{\rm{PWntSC}}}=0.1$, ${k}_{{\rm{PWntTA}}}=0.1,$ ${k}_{{PYAP}}\,$= 0.1, n = 2. a. u.: arbitrary units.

**Fig. 3:**
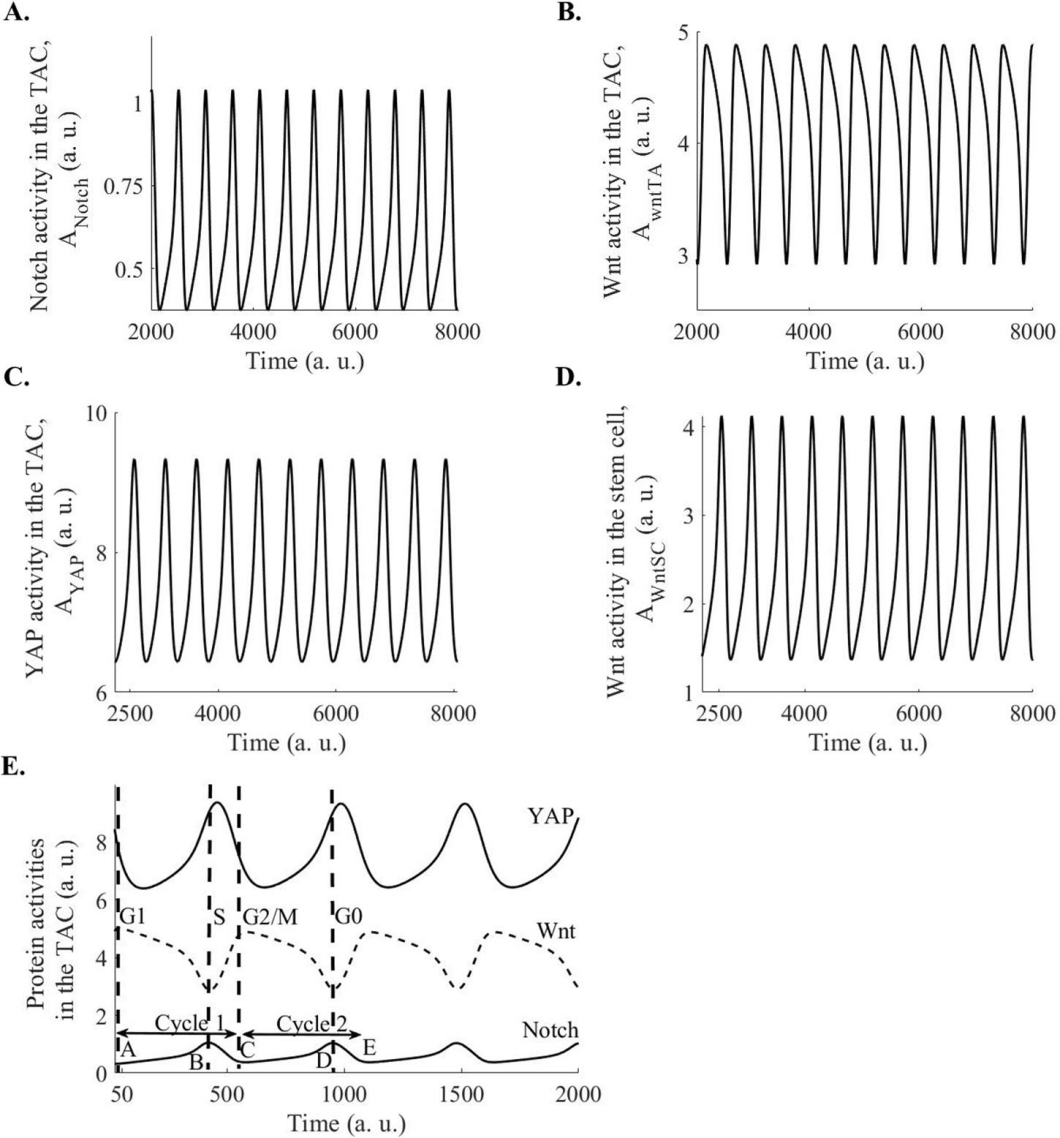
Limit cycle oscillation in the Notch, Wnt, and nuclear YAP/TAZ activities in the TACs, and Wnt activity in the epidermal SCs, which interact with TACs through the Delta-Notch interaction. **A** Limit cycle oscillations in the Notch activity in TACs have been observed. **B** Limit cycle oscillations in the Wnt activity in TACs have been observed. **C** Limit cycle oscillations in the nuclear YAP activity in TACs have been observed. **D** Limit cycle oscillations in the Wnt activity in SCs have been observed. **E** Limit cycle oscillations in the Notch, Wnt, and nuclear YAP activities in the TACs have been observed. The plots have been made for ${k}_{{Notch}}=1$, ${k}_{{\rm{WntSC}}}=1.4,{k}_{{\rm{WntTA}}}=1,\,{k}_{{YAP}}=0.188$, ${k}_{{dNotch}}=0.2$, ${k}_{{\rm{dWntSC}}}=0.2$, ${k}_{{\rm{dWntTA}}}=0.2,$ ${k}_{{dYAP}}=0.02$, ${k}_{{\rm{PWntSC}}}=0.1$, ${k}_{{\rm{PWntTA}}}=0.1,$ ${k}_{{PYAP}}\,$= 0.1, ${k}_{{PNotch}}$ = 0.05, n = 2. a. u.: arbitrary units.

Figure 2A–D is the bifurcation diagrams, in which, between the two Hopf bifurcation points, we observe limit cycle oscillations as ${k}_{{PNotch}}$ is varied. Table 1 summarizes the ranges of parameter values over which the system exhibits oscillations when each parameter is varied individually. Further, Fig. 3A–D shows limit cycle oscillation in Notch, Wnt, and Nuclear YAP activities in TAC and Wnt activity in the SC. In Fig. 3E, we observe that the peak in the Notch activity corresponds to the trough in the Wnt activity in the TAC (vertical dashed line at point B in Fig. 3E). In contrast, we observe another TAC state in which the Notch activity is at the trough while Wnt activity is at the peak (vertical dashed line at point A in Fig. 3E). In the former TAC state (point B in Fig. 3E), in which the Notch activity is high and the Wnt activity is low, the YAP activity is high while in the latter state (point A in Fig. 3E), in which the Notch activity is low and the Wnt activity is high, the nuclear YAP activity is moderate (Fig. 3E). The TAC oscillates between these two states.

**Table 1:** Parameter ranges supporting limit cycle oscillations in the Notch, Wnt, and nuclear YAP activities in the TACs, and the Wnt activity in the SCs, which interact with TACs through the Delta-Notch interaction

Parameter varied	Oscillatory range (a.u.)
*k*_Notch_	0.997–1.121
*k*_WntSC_	1.397–1.506
*k*_WntTA_	0.502–1.008
*k*_YAP_	0.160–0.189
*k*_dNotch_	0.187–0.200
*k*_dWntSC_	0.189–0.200
*k*_dWntTA_	0.199–0.361
*k*_dYAP_	0.020–0.021
*k*_PNotch_	0.050–0.058
*k*_PWntSC_	0.100–0.134
*k*_PWntTA_	0.000–0.106
*k*_PYAP_	0.087–0.100

The ranges of parameter values over which limit cycle oscillations were observed are summarized, where one parameter varied at a time while the other parameters were fixed. The fixed parameter values were ${k}_{{Notch}}=1$, ${k}_{{\rm{WntSC}}}=1.4,\,{k}_{{\rm{WntTA}}}=1,{k}_{{YAP}}=0.188$, ${k}_{{dNotch}}=0.2$, ${k}_{{\rm{dWntSC}}}=0.2$, ${k}_{{\rm{dWntTA}}}=0.2,$ ${k}_{{dYAP}}=0.02$, ${k}_{{\rm{PWntSC}}}=0.1$, ${k}_{{\rm{PWntTA}}}=0.1,$ ${k}_{{PYAP}}$ = 0.1, ${k}_{{PNotch}}$ = 0.05, n = 2. a. u.: arbitrary units.

p73 is an important protein that causes the differentiation of keratinocytes^13,14^. The high and low states of nuclear YAP activity have important implications for the TACs. Blocking the nuclear activity of YAP decreases p73 expression in keratinocytes^15^. Thus, nuclear YAP activity is involved in causing p73 expression. On the other hand, the cytoplasmic YAP binds to p73 and causes cell differentiation^16^. Since the nuclear YAP activity oscillates between a high and a low level in the TAC, the high YAP activity causes p73 expression, while a low nuclear YAP activity, i.e., a high cytoplasmic YAP, causes p73-mediated cell differentiation. Thus, during the whole cycle, YAP acts to cause the differentiation of keratinocytes through p73. Further, due to the critical role of YAP in epidermal differentiation and regeneration, it is not surprising that it has been implicated in the development of multiple skin diseases e.g. Basal cell carcinoma^17,18^, Squamous cell carcinoma^18,19^, Spindle cell carcinoma^19^, Junctional epidermolysis bullosa^20^, Atopic dermatitis^21^, Wound healing^22,23^, and Psoriasis^24^.

### The dynamics of the Notch-Wnt-YAP network under stochastic perturbations

**Fig. 4:**
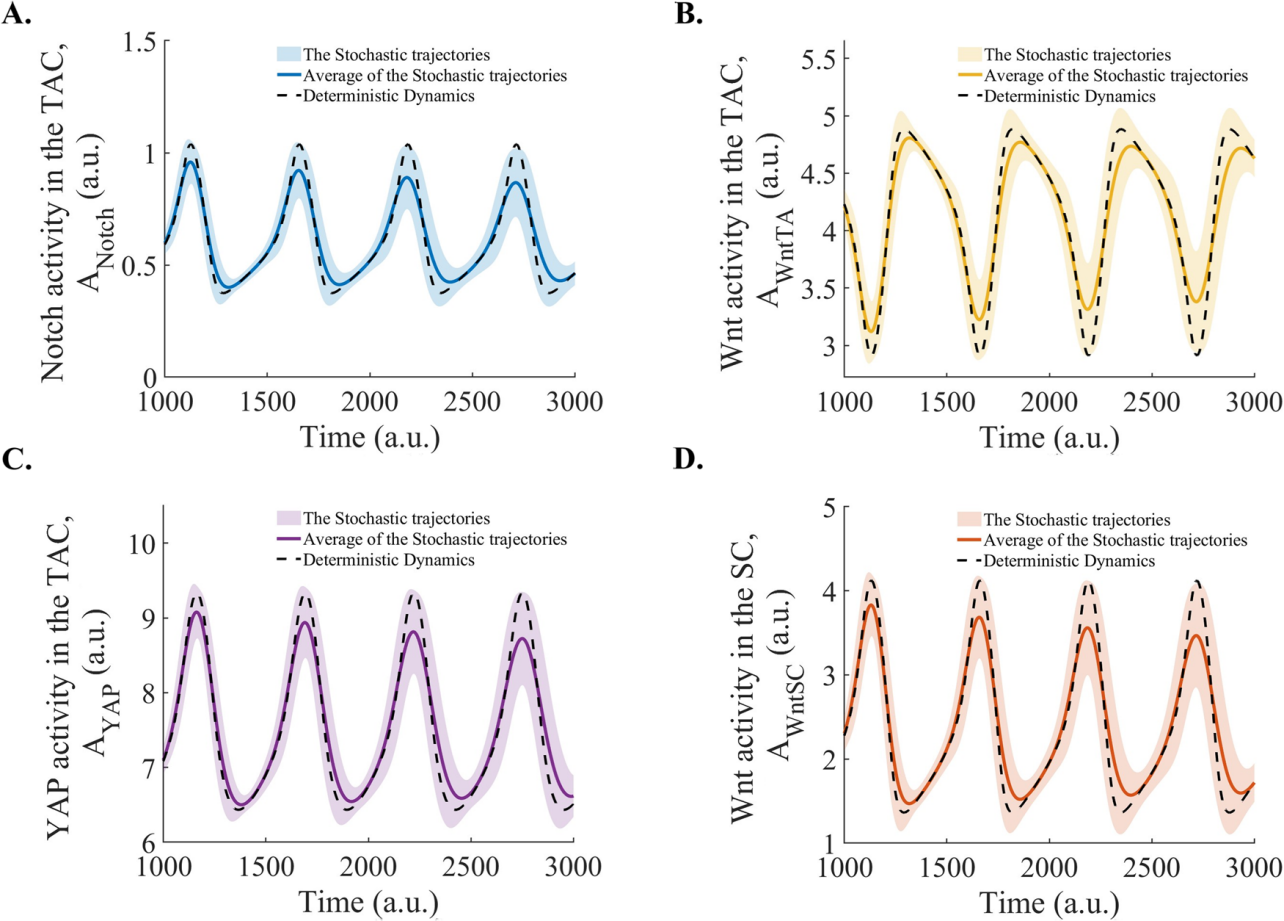
Partial stochastic dynamics of the Notch, Wnt, and nuclear YAP activities in the TACs, and Wnt activity in the SC, modeled using the Euler-Maruyama method. Average and variation of (**A**) The Notch activity in TACs, (**B**) The Wnt activity in TACs, (**C**) The nuclear YAP activity in the TACs, and (**D**) The Wnt activity in the SCs, with a random noise, compared to their deterministic dynamics. The shaded regions show the range of activities across the 1000 stochastic trajectories, while the solid lines represent the stochastic mean, and dashed lines are the deterministic dynamics. The simulations were performed with parameter values: ${k}_{{Notch}}=1$, ${k}_{{\rm{WntSC}}}=1.4,\,{k}_{{\rm{WntTA}}}=1,{k}_{{YAP}}=0.188$, ${k}_{{dNotch}}=0.2$, ${k}_{{\rm{dWntSC}}}=0.2$, ${k}_{{\rm{dWntTA}}}=0.2,$ ${k}_{{dYAP}}=0.02$, ${k}_{{\rm{PWntSC}}}=0.1$, ${k}_{{\rm{PWntTA}}}=0.1,$ ${k}_{{PYAP}}\,$= 0.1, ${k}_{{PNotch}}$ = 0.05, n = 2. a. u.: arbitrary units.

Next, we investigated whether these limit cycle oscillations persist under molecular noise. Figure 4A–D shows that the mean trajectories from the stochastic simulations remain close to the deterministic dynamics, and the range of variation in the trajectories is relatively narrow. This demonstrates the robustness of the oscillatory dynamics in Notch, Wnt, and Nuclear YAP activities in the TACs and Wnt activity in the SC under stochastic perturbations, indicating that the epidermal regulatory network can sustain the oscillatory behavior despite the inherent stochasticity in the system. The stochastic mean deviates from deterministic dynamics more at the maxima and minima, reflecting the accumulations of the stochastic fluctuations in the near-zero slope regions. Nonlinear stochastic systems remain at extrema for a longer period due to the zero slope, allowing asymmetrically distributed perturbations to amplify, thereby shifting the stochastic mean away from the deterministic path. This behaviour is consistent with large deviation theory, which explains that rare but significant fluctuations can dominate system behaviour near extrema.

### Oscillation vs. bistability in the Notch, Wnt, and YAP activities

**Fig. 5:**
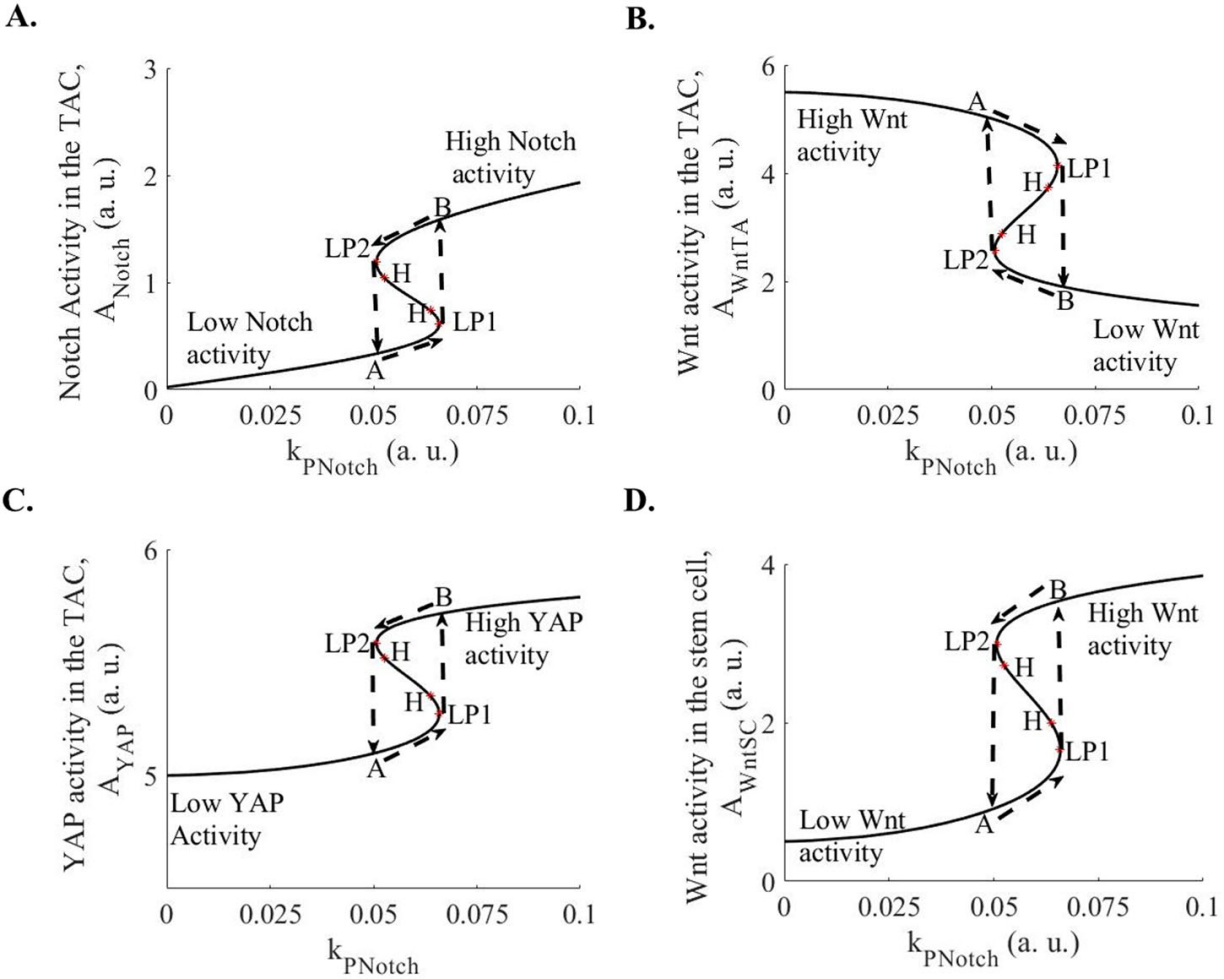
Bistability in the Notch, Wnt, and nuclear YAP/TAZ activities in the TACs, and Wnt activity in the epidermal SCs, which interact with the TACs through the Delta-Notch interaction. **A** A bifurcation diagram in the Notch activity in the TACs as a function of generation of the Notch activity, the parameter k_PNotch_, in the TACs, has been shown. Between the two loop points, LP1 and LP2, the steady states are unstable. All other steady states are stable. **B** A bifurcation diagram in the Wnt activity in TACs as a function of the generation of the Notch activity, the parameter k_PNotch_, in TACs, has been shown. Between the two loop points, LP1 and LP2, the steady states are unstable. All other steady states are stable. **C** A bifurcation diagram in the nuclear YAP activity in the TACs as a function of generation of the Notch activity, the parameter k_PNotch_, in TACs, has been shown. Between the two loop points, LP1 and LP2, the steady states are unstable. All other steady states are stable. **D** A bifurcation diagram in the Wnt activity in SCs as a function of the generation of the Notch activity, the parameter k_PNotch_, in TACs, has been shown. Between the two loop points, LP1 and LP2, the steady states are unstable. All other steady states are stable. The plots have been made for ${k}_{{Notch}}=1$, ${k}_{{\rm{WntSC}}}=0.85,\,{k}_{{\rm{WntTA}}}=1,{k}_{{YAP}}=0.02$, ${k}_{{dNotch}}=0.2$, ${k}_{{\rm{dWntSC}}}=0.2$, ${k}_{{\rm{dWntTA}}}=0.2,$ ${k}_{{dYAP}}=0.02$, ${k}_{{\rm{PWntSC}}}=0.1$, ${k}_{{\rm{PWntTA}}}=0.1,$ ${k}_{{PYAP}}\,$= 0.1, n = 2. a. u.: arbitrary units.

For a different set of parameter values, we observe bistability in the activities of the three proteins as the generation of the Notch activity, ${k}_{{PNotch}}$, is varied (Fig. 5A–D). Figure 5A plots the steady state of Notch activity as a function of the parameter, ${k}_{{PNotch}}$. Two kinds of steady states are observed in Fig. 5A: (1) Low Notch activity steady states and (2) High Notch activity steady states. Between two loop points (LP1 and LP2), the steady states are unstable while all other steady states are stable (Fig. 5A). As the parameter, ${k}_{{PNotch}}$ is increased, the cell state moves on the low-notch activity path till LP1 (Fig. 5A). A further increase in ${k}_{{PNotch}}$ causes a jump in the Notch activity to the high-notch activity path (Fig. 5A). Once on the high-notch activity pathway, decreasing ${k}_{{PNotch}}$ does not cause an immediate jump back to the low-notch activity path, and the cell state remains on the high-notch activity path until the loop point 2 (LP2) is reached. A further decrease causes the jump in the steady state to the low-notch activity path. Thus, the Notch activity shows hysteresis and bistability. Similarly, Wnt and YAP activities in the TAC and Wnt activity in the SC show bistability (Fig. 5B–D).

However, the bistable system is an incomplete mechanism because it does not explain how the TACs, which interact with SCs, differentiate. In contrast, in the oscillatory system, the low nuclear YAP activity (i.e., the high cytoplasmic YAP activity) causes cell differentiation by binding with p73^16^, while the high nuclear YAP activity causes p73 transcription^15^, which explains the differentiation process as discussed in the discussion section.

### What may happen after a few cycles of oscillations in Notch, Wnt, and nuclear YAP activities in the TAC interacting with a stem cell

**Fig. 6:**
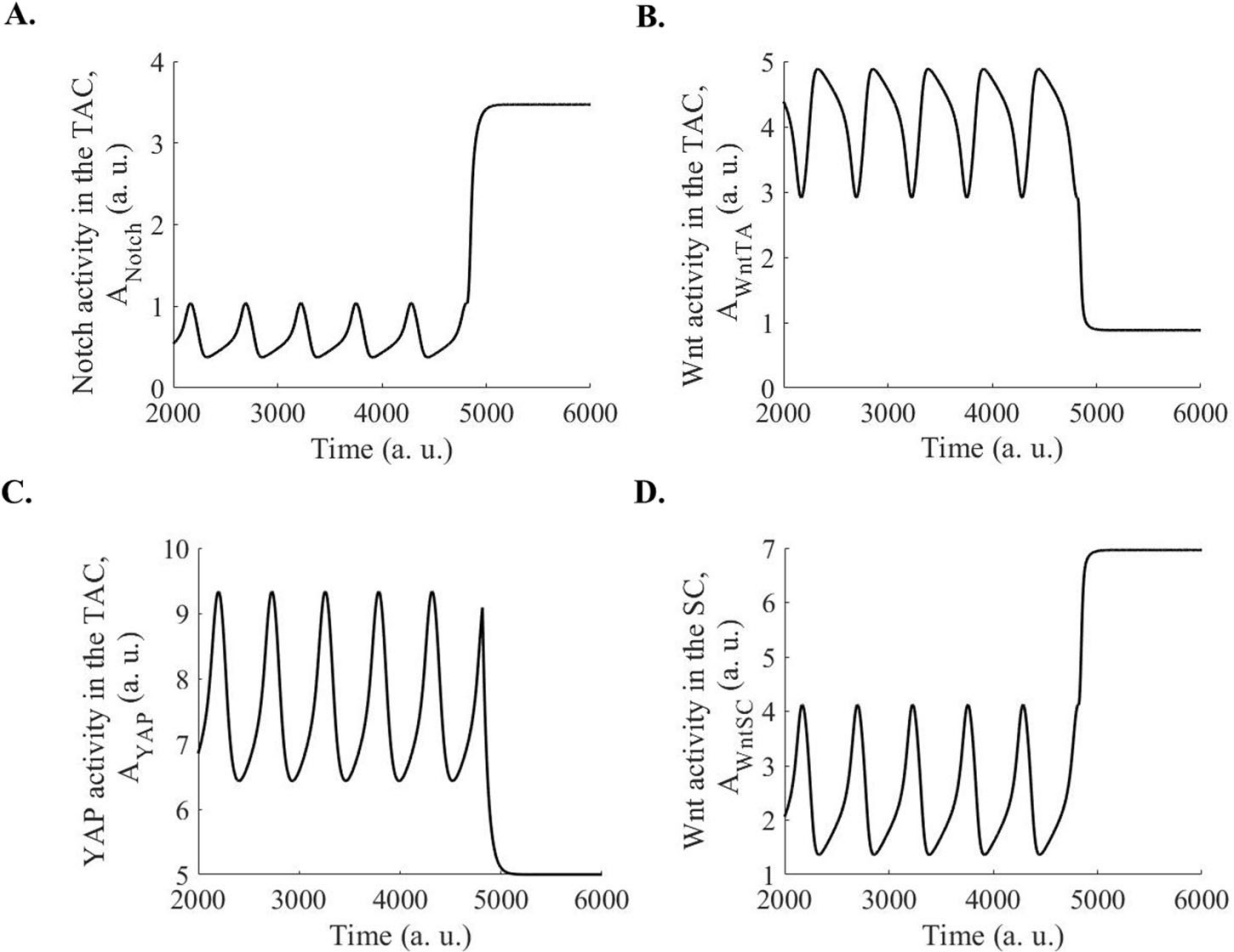
Few cycles of oscillations in the Notch, Wnt, and nuclear YAP/TAZ activities in the TACs cause cell cycle progression and terminal differentiation of TACs. **A** Few cycles of oscillations in the Notch activity in the TAC and Notch activity in the TDCs have been shown. After terminal differentiation of TACs, the regulation of nuclear YAP activity in response to Notch activity has been ablated. Then, the oscillation in the Notch activity in the TACs stops, and the Notch activity attains a high value. **B** Few cycles of oscillations in the Wnt activity in the TAC and Wnt activity in the TDCs have been shown. After terminal differentiation of TACs, the regulation of nuclear YAP activity in response to Notch activity has been ablated. Then, the oscillation in the Wnt activity in the TACs stops, and the Wnt activity attains a low value. **C** Few cycles of oscillations in the YAP activity in the TAC and YAP activity in the TDCs have been shown. After terminal differentiation of TACs, the regulation of nuclear YAP activity in response to Notch activity has been ablated. Then, the oscillation in the YAP activity in the TACs stops, and the YAP activity attains a low value. **D** Few cycles of oscillations in the Wnt activity in the SC have been shown. After terminal differentiation of TACs, the regulation of nuclear YAP activity in response to Notch activity in the TAC has been ablated. Then, the oscillation in the Wnt activity in the SC stops, and the Wnt activity attains a high value. The plots have been made for ${k}_{{Notch}}=1$, ${k}_{{\rm{WntSC}}}=1.4,{k}_{{\rm{WntTA}}}=1,{k}_{{YAP}}=0.188$, ${k}_{{dNotch}}=0.2$, ${k}_{{\rm{dWntSC}}}=0.2$, ${k}_{{\rm{dWntTA}}}=0.2,$ ${k}_{{dYAP}}=0.02$, ${k}_{{\rm{PWntSC}}}=0.1$, ${k}_{{\rm{PWntTA}}}=0.1,$ ${k}_{{PYAP}}\,$= 0.1, ${k}_{{PNotch}}$ = 0.05, n = 2. a. u.: arbitrary units.

After a few cycles of oscillations, the TACs terminally differentiate, and cells move to the suprabasal layer, losing integrin signaling. Further, YAP is translocated to the nucleus in response to the integrin signaling, and in differentiated keratinocytes, YAP is in the cytoplasm^8^ due to a lack of integrin signaling in the suprabasal layer. Further, the nuclear translocation of YAP in response to Notch activity involves integrins^25^. Thus, after differentiation, we have removed the regulation of nuclear YAP activity in response to the Notch activity in our model. Figure 6A–D shows the Notch, the Wnt, and the nuclear YAP activities in the TACs and SCs. In TDCs, oscillations cease, and Notch activity attains a high value (Fig. 6A), keeping the cells out of the cell cycle through upregulation of p21. In contrast, the Wnt and nuclear YAP activities attain low values (Fig. 6B, C). Wnt and YAP activities are also no longer required for the progression of the cell cycle and differentiation. Further, the Wnt activity in the SC, which interacted with the TAC, attains a high value (Fig. 6D).

## Discussion

The oscillation observed in the activities of Wnt, Notch, and YAP pathways in the TACs has important implications for cell cycle progression and differentiation of TACs. In this context, Wnt activity causes the transcription of cyclin D and E and, thus, is required for the progression of the G1 phase of the cell cycle^26^. Wnt activity induces c-myc, which upregulates cyclin D and represses p21/p27^26^. Similarly, Wnt activity is also required for the progression of the G2 and M phases of the cell cycle^26^. On the other hand, Notch activity upregulates p21^27^, causing growth arrest. We considered two cycles of Notch, Wnt, and nuclear YAP activities in the TAC: Cycle 1 is from point A to B and B to C, and cycle 2 is from C to D and D to E in Fig. 3E. In the first half of the first Wnt cycle, at point A, when Wnt activity reaches a peak in the TAC, the Notch activity reaches a trough (Fig. 3E), causing G1 phase progression and entry into the S-phase of the cell cycle in the TACs interacting with the SCs. In the first half of the first cycle, between points A and B, nuclear YAP also reaches a low value (Fig. 3E), increasing the cytoplasmic YAP, which can cause differentiation through binding with p73^16^ but cells do not differentiate because p73 has not been transcribed yet, since the p73 transcription requires high nuclear YAP^15^. Then, in the second half of the first cycle (between points B and C in Fig. 3E), the nuclear YAP activity reaches a high value (Fig. 3E), causing p73 transcription. Further, nuclear YAP activity is required for proper progression in S-phase and genomic integrity^28,29^. Thus, in this part, due to nuclear YAP activity, cells progress through the S-phase (between points B and C in Fig. 3E). Then, the second cycle starts, and Wnt activity reaches the peak at point C, causing progression through the G2 and M phases. The nuclear YAP reaches a low value or the cytoplasmic YAP reaches a high value between points C and D. Since p73 has already accumulated due to the high nuclear YAP in the second half of the previous cycle (between points B and C in Fig. 3E), the high cytoplasmic YAP or low nuclear YAP between points C and D (Fig. 3E) initiates the terminal differentiation program through p73. Then, at point D, Notch activity reaches a peak (Fig. 3E), causing asymmetric cell division and mediating epidermal differentiation^4^. Further, the peak of Notch activity upregulates p21, causing the cell cycle exit of the TDCs. Thus, coordination among the three proteins, Wnt, Notch, and YAP, plays critical roles in epidermal differentiation.

The effect of Wnt, YAP, and Notch activities on effector proteins that control cell cycle, e.g., cyclin D, p21, and that control epidermal differentiation, e.g., p73, can be integrated into our model as the downstream targets of Wnt, YAP, and Notch activities, and the model can be coupled to the cell cycle. Thus, an improved model that integrates Wnt, YAP, and Notch activities, their downstream targets, and the cell cycle can be produced, whose output would be the production of TDCs from the interaction of SCs and TACs. Further, when a TAC differentiates, the TDC moves to the suprabasal layer. Thus, a switch in the microenvironment of the cell occurs. An improved model that incorporates not only the above reactions but also the effect of extracellular matrix, e.g., integrin signaling, on Wnt, Notch, and YAP pathways will make the differentiation process an emergent property of SC-TAC interaction.

Wnt activity causes Delta and DLL1 transcription^30^. Similarly, YAP/TAZ causes the expression of Delta and DLLs^6^. The Wnt, Notch, and YAP/TAZ activities in the TACs, and the Wnt activity in SC are all interrelated via the feedback loops. For example, Wnt activity in SC causes the expression of notch ligand Delta on the surface of SCs^30^, which activates the Notch pathway in the TACs that interact with SCs through Notch-Delta interaction. The consequent Notch activity in the TACs suppresses the Wnt activity in these cells, decreasing the expression of Delta by the TACs^31,32^. The lower expression of Delta by the TACs causes lower-notch activity in the interacting SC. Further, the lower notch activity in SC causes higher Wnt activity in these cells^31^ causing the higher expression of Delta by these cells, which causes the high, or a peak, of the Notch activity in the interacting TACs. On the other hand, the notch activity in TAC causes the translocation of YAP/TAZ to the nucleus^25^. The nuclear YAP/TAZ causes the transcription of Delta on the surface of TACs. The Delta on the surface of TACs inhibits the Notch receptor on the surface of these cells through the ‘in *cis*’ inhibition^6^, decreasing the Notch activity in these cells and causing a trough of the Notch activity in TACs. Thus, the interplay of the positive and negative feedback loops is responsible for the oscillations in the activities of these pathways.

Oscillations can occur in a single-delayed negative feedback loop^33^. However, in cell differentiation, limit cycle oscillations may occur so that the oscillations do not dampen out before the terminal differentiation occurs. For limit cycle oscillations in a single negative feedback loop, the Hill coefficient should be larger than 8^34^, a value unrealistic in biological systems^33^. On the other hand, a high value of the Hill coefficient widens the parameter space of oscillations^35,36^. Further, although a high value of the Hill coefficient is unrealistic in biological systems, it is desirable for observing oscillations in a wider parameter space. In biological systems, positive feedback loops are commonly found to be coupled with a negative feedback loop^37^. In our system, the ultrasensitivity introduced by the coupling of the two positive feedback loops with a negative feedback loop may widen the parameter space of observing the limit cycle oscillation. Further, the coupling of multiple positive feedback loops cumulatively decreases the cooperativity needed to observe oscillations^37^. Thus, coupling of positive feedback loops with a negative feedback loop may have evolved to facilitate oscillations at lower, kinetically feasible, degrees of cooperativity^37^. Furthermore, unlike in the cell population dynamics, in molecular reactions, the observed input-output patterns differ from Michaelis-Menten kinetics^38^. Specifically, to increase the robustness of the response and filter out noise in molecular reactions, the output is weakly sensitive to changes in the low-intensity inputs^38^. Weak sensitivity at low input implies that the output varies in proportion to log(input), observed in the Hill function for small input values, rather than linearly described by Michaelis-Menten kinetics^38^. In contrast, the fluctuations in cell number density generated by the noise in the input, caused by the high sensitivity of the output, due to the use of the Michaelis-Menten equation in the cell population dynamics, may not be as detrimental to biological systems as the fluctuations in the output of the molecular reactions. Thus, we used Michaelis-Menten kinetics for cell population dynamics and Hill function with the lowest and biologically realistic Hill coefficient, n = 2, in the dynamics of Wnt, Notch, and YAP activities.

An alternative model of epidermal differentiation exists. In the alternative model, basal cells secrete both Wnt ligands and a Wnt inhibitor, Dkk, due to autocrine Wnt signaling^3,9^. Differential diffusion of Wnt ligands and Dkk may restrict Wnt signaling to the basal layer, while cells leaving the basal layer encounter an increased amount of Dkk and differentiate^3,9^. Thus, the alternative model is based on the spatial gradient of Wnt signaling strength, while the model given in this paper is based on temporal dynamics of the three pathways. Further, the alternative model does not account for Notch signaling, which has an important role in epidermal differentiation^12,39^. Furthermore, it has been shown that asymmetric cell division, regulated by proteins asymmetrically distributed at the cell cortex during mitosis, causes epidermal differentiation, and asymmetric cell division and Notch signaling are linked in this process^4^. Thus, while spatial differences in the extracellular matrix and activities of signaling pathways may be responsible for the differences among the cells of different layers of the differentiated suprabasal layers, the asymmetric cell division and involvement of the cell cycle support our temporal model.

In agreement with a previous study^12^, our paper suggests that epidermal stemness is functionally related to the expression of the Notch ligand, Delta, by the epidermal stem cells. Epidermal stem cells are those cells in the basal layer that can induce the terminal differentiation of the TACs. Based on this definition, we show that the SCs must express Delta higher than a critical value, ${d}_{{Cr}}$ (Eq. 5):

$${{\rm{d}}}_{{\rm{Cr}}}=\frac{{\rm{Rate\; of\; loss\; of\; the\; TDCs}}}{{\rm{Rate\; of\; generation\; of\; the\; TDCs}}}$$

Thus, ${d}_{{Cr}}$ is related to the rate of turnover of the epidermis, and the turnover rate of the differentiated epidermis controls whether a basal keratinocyte is a SC or a TAC. If the rate of generation of the TDCs is high, the ${d}_{{Cr}}$ will be low. Thus, the processes that accelerate the differentiation of TACs lower the threshold of Delta expression required for the cells to function as epidermal stem cells. Since TACs differentiate following the completion of the cell cycle while interacting with the stem cell, the denominator and, thus, ${d}_{{Cr}}$ depends on the rate of the cell cycle. Further, since the S-phase of the cell cycle occurs when NADH/NAD+ ratio is the highest, and since NADH/NAD+ is linked to the circadian clock^7,40^, ${d}_{{Cr}}$ is linked with the rate of the circadian clock. On the other hand, in the numerator of ${d}_{{Cr}}$ equation, the rate of loss of the TDCs, is normally linked to environmental conditions and the rate of Notch activity-independent apoptosis. When the above equation of ${d}_{{Cr}}$ is not satisfied by the epidermis, the epidermal turnover is disturbed, causing skin diseases. Further, since Wnt and YAP activities cause Delta expression in the cells^6,30^, these two pathways are important for epidermal stemness.

Previously, it has been shown that in epidermal stem cells, NADH concentration oscillates along with the circadian rhythm, and in S-phase, NADH concentration is the highest, so that during this phase the cell is in the least oxidative state, minimizing DNA damage during the DNA synthesis^7,40^. Further, the S-phase happens during the night time^40^. Thus, the cell cycle takes approximately 24 h. Since differentiation requires the cell cycle, which has been linked to the circadian clock^7^, the period of cyclic variation in Wnt, Notch, and YAP activities is linked to the circadian clock. In our model, two cycles of the Notch, Wnt, and YAP activities are required for one cell cycle and differentiation. Thus, the period of oscillations in Notch, Wnt, and YAP activities is approximately 12 h.

LGR6 and Lrig1 have been discovered as stem cell markers^10^ and represent two distinct epidermal stem cells in the basal layer of the epidermis^41^. LGR6 is an enhancer of Wnt signaling and forms a positive feedback loop with the Wnt pathway^42^. Thus, LGR6 may be responsible for increased expression of the Notch ligand, Delta, required for epidermal cells to function as stem cells. On the other hand, Lrig1 represents quiescent stem cells^41^. However, Lrig1 cells become primed to proliferate under BMP or FGF treatment and proliferate in the presence of EGF/FGF^11^, the growth factors present in the epidermis. Thus, while LGR6 and Lrig1 are markers of epidermal stem cells, Delta expression by LGR6 cells has functional relevance for the epidermis. Further, Clayton et. al showed that a single type of progenitor cell maintains the normal epidermis^43^. Similarly, Lim et. al showed that the majority of basal cells express Axin2, a Wnt target gene^3^. In the oscillating system presented here, all states of TACs are linked and realized periodically.

In summary, we presented detailed insights into epidermal stemness and differentiation processes, giving models using mathematical and systems biology approaches. Our models and analyses may have general implications for stemness and differentiation of other tissues.

## Methods

### Model of epidermal stemness

**Fig. 7:**
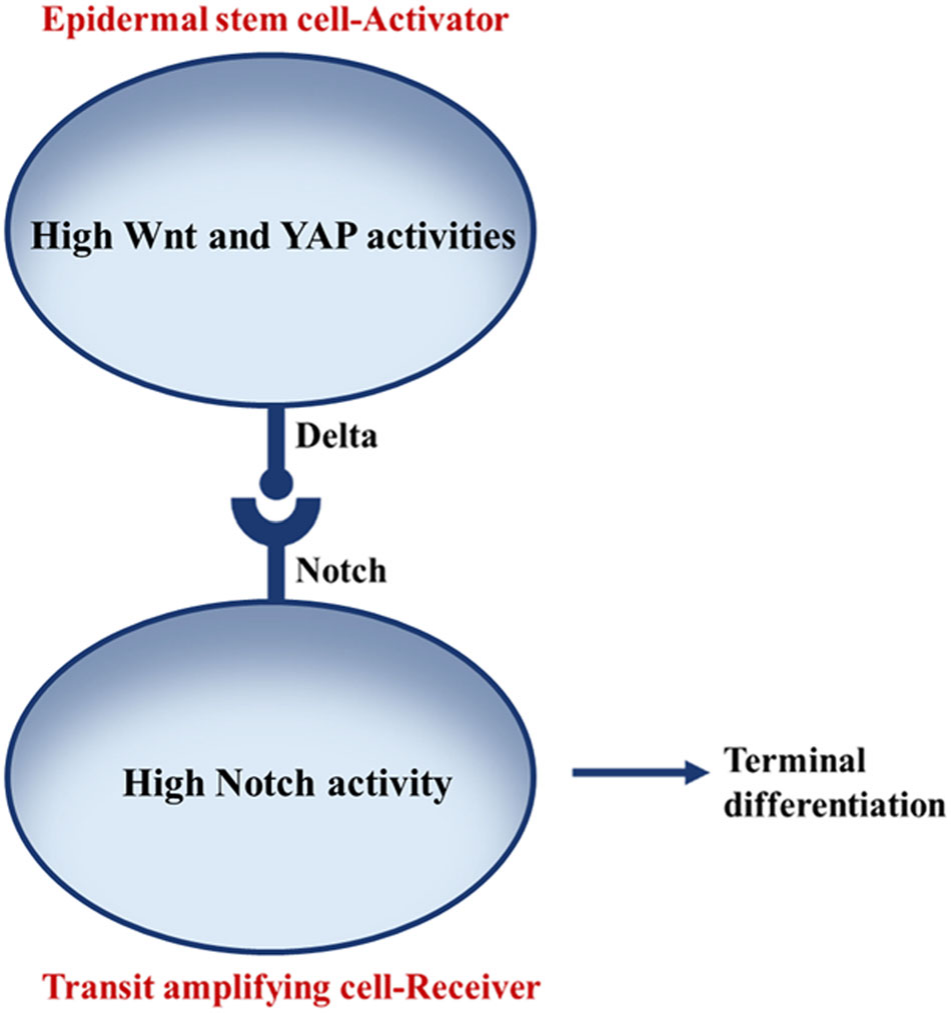
Expression of Notch ligand, Delta, has functional relevance for epidermal SCs and differentiation of the TACs. Delta-Notch interaction between epidermal SCs, expressing Delta, and the TACs, expressing Notch, has been shown. Through Delta-Notch interaction, SCs induce the terminal differentiation of TACs.

Based on a study by Lowell et al^12^, we define a basal keratinocyte as an epidermal SC if it can cause differentiation in a TAC, interacting with the SC through Delta-Notch interaction, regenerating the epidermis. Wnt pathway activator, LEF1, was found to bind multiple sites in the promoter of DLL1, a ligand of the Notch pathway^30^. Further, the induced expression of LEF1–β–catenin or Wnt-1 caused the expression of endogenous DLL1 in the cells^30,44^. Thus, the high Wnt activity in epidermal SCs leads to the high expression of DLL, which activates the Notch signaling in the TAC (Fig. 7) and asymmetrically induces the terminal differentiation of the progeny.

In this model, an epidermal SC, expressing high levels of Delta/DLL induces the Notch pathway in an adjacent TAC (Fig. 7). We denote the number density of the terminally differentiated progeny that are produced in the basal layer due to delta-notch interaction as C_TDC,_ and the concentration of the Notch ligands, Delta/DLL, on the SC surface as d. Each time a TAC that interacts with an SC through Notch-Delta interaction divides asymmetrically, it produces one TAC and one TDC in the basal layer. Thus, the TAC interacting with an SC self-renews itself, and the production rate of TDCs is the same as that of TACs that interact with an SC. Further, in the cell division, the parent TAC ceases to exist, and the daughter TDC moves to the suprabasal layer. Thus, the rates of loss of the TDCs and TACs in the basal layer are also the same. Since, in the basal layer, both the rate of production and the rate of loss of TDCs are the same as those of the TACs, respectively, the number density of TACs that productively produce TDCs and the number density of daughter TDCs in the basal layer are the same, maintaining the invariance and the homeostasis of this layer. Some TACs do not interact with stem cells and do not give rise to a TDC, as those TACs are not located at the boundary of the epidermal stem cell cluster. Those TACs are not part of our model. When the number density of TACs that interact with an SC through the Delta-Notch interaction and produce TDCs is large, the production rate of TDCs is expected to be independent of the number density of TACs since they are in excess. On the other hand, when this number density is small, the rate of production of TDCs is expected to depend on certain powers, or cooperativity, of the number density of TACs and the expression level of the notch ligand, Delta (d), by the epidermal SCs. The feasibility of a biological process decreases as cooperativity, which increases the complexity of the system, increases^33,37^. Since one cell divides into two cells in the cell division, the cooperativity is not expected in this process and is assumed to be 1. Then, the rate of production of the TDCs in the basal layer can be given following Michaelis-Menten kinetics, where the concentration of DLL takes the place of an enzyme, and the number density of TACs (=${{\rm{C}}}_{{\rm{TDC}}}$) that interact with an SC and produce TDCs takes the place of the substrate. Thus, the net rate of increase in the number density of TDCs in the basal layer, $\frac{{{\rm{dC}}}_{{\rm{TDC}}}}{{\rm{dt}}}$, is given as:

$$\frac{{\mathrm{dC}}_{\mathrm{TDC}}}{\mathrm{dt}}=\frac{{{\rm{k}}}_{1\,}{{\rm{C}}}_{\mathrm{TAC}}\,{\rm{d}}}{{{\rm{k}}}_{2}+{{\rm{C}}}_{\mathrm{TAC}}}-{{\rm{k}}}_{{\rm{d}}1}{{\rm{C}}}_{\mathrm{TDC}}$$

or,

6 $$\frac{{\mathrm{dC}}_{\mathrm{TDC}}}{\mathrm{dt}}=\frac{{{\rm{k}}}_{1\,}{{\rm{C}}}_{\mathrm{TDC}}\,{\rm{d}}}{{{\rm{k}}}_{2}+{{\rm{C}}}_{\mathrm{TDC}}}-{{\rm{k}}}_{{\rm{d}}1}{{\rm{C}}}_{\mathrm{TDC}}$$

or,

7 $$\frac{{{\rm{dC}}}_{{\rm{TDC}}}}{{\rm{dt}}}=\left(\frac{{{\rm{k}}}_{1}{\rm{d}}}{{{\rm{k}}}_{2}+{{\rm{C}}}_{{\rm{TDC}}}}-{{\rm{k}}}_{{\rm{d}}1}\right){{\rm{C}}}_{{\rm{TDC}}}$$

where, k_1_, k_2_, and k_d1_ are parameters, C_TDC_ is the number density of the TDCs generated from the TACs in the asymmetric cell divisions, and d is the concentration of the Delta/Delta-like ligand (DLL) on the surface of the epidermal stem cell in the basal layer. The parameter k_1_ is the rate constant with which the epidermal stem cell causes the production of the TDCs from the TACs by activating the Notch pathway. The parameter k_2_ represents the fold decrease in the rate of production of TDCs due to the Notch activity-independent apoptosis, while in ${k}_{2}+{C}_{{TDC}}$, i.e., in the denominator of the first term in Eq. 7, C_TDC_ represents the fold decrease in the rate of production of the TDCs due to the Notch-activity-dependent apoptosis of the TDCs. The Notch activity-dependent apoptosis of TDCs will be effective when ${C}_{{TDC}}$ is comparable to ${k}_{2}$, i.e., when Notch pathway activity is high and the magnitude of ${C}_{{TDC}}$ is high enough. Further, after their differentiation, the TDCs move up in the suprabasal layers. The parameter k_d1_ represents the rate constant with which the TDCs move up in the suprabasal layer. We have modeled the production of TDCs in the basal layer. In our model, the loss rate of TDCs from the basal to suprabasal layer is ${{\rm{k}}}_{{\rm{d}}1}{{\rm{C}}}_{{\rm{TDC}}}$ in Eq. 6. Thus, since ${{\rm{C}}}_{{\rm{TDC}}}$ is notch activity dependent, so is the loss rate of TDCs from the basal to the suprabasal layer. Further, although we have not modeled the suprabasal layers, in the suprabasal layers, notch activity promotes the formation of spinous and granular layers in a Hes1- and Ascl2-dependent manner, promoting dynamics and stratification of the suprabasal layers^45^.

### Model of epidermal differentiation

**Fig. 8:**
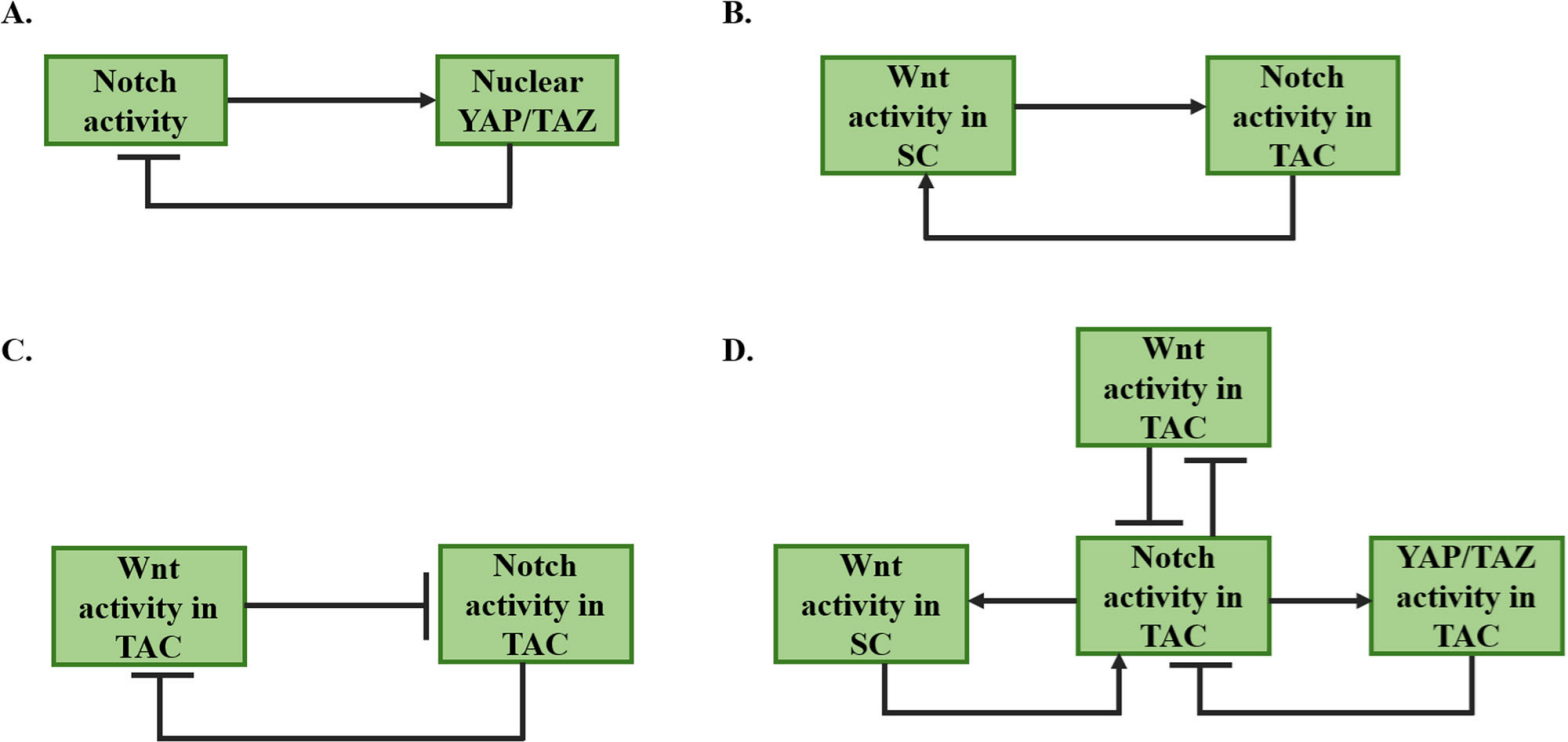
Regulation of Notch, Wnt, and YAP activities by feedback loops. **A** A negative feedback loop between Notch activity and nuclear YAP/TAZ activity in the TACs^6,25^, which interact with the epidermal SCs through the Delta-Notch interaction. **B** A positive feedback loop between Wnt activity in SCs and Notch activity in TACs^30–32^, which interact with SCs through the Delta-Notch interaction. **C** A double-negative feedback loop between the Wnt and the Notch activities in TACs, which interact with SCs through the Delta-Notch interaction^31^. **D** Combining the feedback loops in (**A**, **B**, and **C**), we get interacting positive and negative feedback loops among the Wnt, the Notch, and the nuclear YAP activities in the TACs^6,25,30–32^, which interact with SCs through the Delta-Notch interaction.

Epidermal SCs activate the Notch pathway in the TACs that lack p53, causing nuclear translocation of YAP/TAZ^25^, which in turn blocks the Notch pathway^6^ in the TACs. Thus, Notch and YAP/TAZ form a negative feedback loop in the TACs (Fig. 8A). In the normal epidermis, p53 expression was not observed, while PLK4 overexpression stabilized p53 and caused its expression in around 25% of the basal keratinocytes^46^. Further, p53 stabilization in the epidermis due to PLK4 overexpression caused a differentiation defect^46^.

Further, the high Wnt activity in epidermal SCs causes DLL expression^30^ in these cells, and activates the Notch pathway in the TACs, interacting with the epidermal stem cells. Thus, high Wnt activity in SC causes high Notch activity in the TAC (the forward arm of the positive feedback loop in Fig. 8B). On the other hand, the high Notch activity in the TAC causes low Wnt activity^31^ and low expression of Notch ligands by these cells^32^, thus, causing low Notch activity in the SC, interacting with the TAC. Since the Notch and the Wnt activities in the same cell negatively regulate each other^31^, the low-notch activity in the SC causes high Wnt activity in these cells. Thus, high Notch activity in TAC causes high Wnt activity in the SC (the backward arm of the positive feedback loop in Fig. 8B). Therefore, there is a positive feedback loop between the Notch activity in the TACs and the Wnt activity in the epidermal SCs (Fig. 8B). In contrast, both the Notch and the Wnt activities in the same cell negatively regulate each other^31^ (Fig. 8C). Thus, between the interacting SCs and TACs, there are coupled negative and positive feedback loops (Fig. 8D, which is based on Fig. 8A–C), which may be responsible for a rich dynamics of Notch, Wnt, and YAP activities in the SCs and TACs.

### Model SC-TAC interaction

In the rate equation of the activity of each protein, i.e., Notch, Wnt, and nuclear YAP, there are three terms: (1) Regulation term (2) Degradation term (3) Generation term. The regulation, positive or negative, is modeled by a Hill function. The degradation term is given as a first-order rate expression. The generation term is due to the production of ligand and/or the receptor of the pathway through a mechanism other than what is involved in the primary regulation term considered in the model. In the case of the Notch activity in the TAC, the Notch receptor is produced by CtIP/CtBP^47^, which also regulates the Notch and Wnt activities through the corresponding repressor complexes^48^. Due to the involvement of this additional mechanism, the generation of Notch activity is considered a variable parameter since CtIP/CtBP also controls the Wnt and the Notch activities, which are variables in the model. In contrast, the generation terms in the Wnt and YAP activities are assumed constant since the concentrations of the ligands/receptors of these pathways are not known to vary due to any other additional mechanisms that interact with the feedback loops other than those already considered in the regulation term.

### Model of the Notch activity in the TAC interacting with the SC

The regulation term in the rate of change of Notch activity, A_Notch_, in the TAC, is given by a Hill function. A Hill function is written in a general form as:

$${{\rm{k}}}_{1}\left(\frac{{{{\rm{X}}}_{{\rm{P}}}}^{{\rm{n}}}}{{{\rm{K}}}^{{\rm{n}}}+{{{\rm{X}}}_{{\rm{P}}}}^{{\rm{n}}}+\sum _{i}{{{\rm{X}}}_{{\rm{Ni}}}}^{{\rm{n}}}}\right)$$

where, ${{\rm{k}}}_{1}$ and K are parameters, and n is the Hill coefficient. X_P_ is the concentration of the positive regulator and X_Ni_ is the concentration of the i^th^ negative regulator. ∑ denotes the summation.

Dividing the above expression by ${{\rm{K}}}^{{\rm{n}}}$,

$${{\rm{k}}}_{1}\left(\frac{{\left({{\rm{X}}}_{{\rm{P}}}/K\right)}^{{\rm{n}}}}{1+{\left({{\rm{X}}}_{{\rm{P}}}/K\right)}^{{\rm{n}}}+\sum _{i}{\left({X}_{{Ni}}/K\right)}^{n}}\right)$$

We denote ${{\rm{X}}}_{{\rm{P}}}/K$ and ${X}_{{Ni}}/K$ as the normalized activities, ${{\rm{A}}}_{{\rm{i}}}$. The Notch activity in the TAC, A_Notch,_ is positively regulated by the Wnt activity in the SC, ${{\rm{A}}}_{{\rm{WntSC}}}$, and negatively regulated by the nuclear YAP activity, ${{\rm{A}}}_{{\rm{YAP}}}$, and Wnt activity in the TAC, ${{\rm{A}}}_{{\rm{WntTA}}}$ (Fig. 8D). Thus, the regulation term in the rate of change of Notch activity, A_Notch_, in the TAC is given as,

$${{\rm{k}}}_{{\rm{Notch}}}\left(\frac{{{{\rm{A}}}_{{\rm{WntSC}}}}^{{\rm{n}}}}{1+{{{\rm{A}}}_{{\rm{WntSC}}}}^{{\rm{n}}}+{{{\rm{A}}}_{{\rm{YAP}}}}^{{\rm{n}}}{{+{\rm{A}}}_{{\rm{WntTA}}}}^{{\rm{n}}}}\right)$$

where, ${{\rm{k}}}_{{\rm{Notch}}}$ is a parameter, and n is the Hill coefficient. Thus, incorporating the three terms, (1) Regulation term, (2) Degradation term (3) Generation term, in the equation of the rate of change in the Notch activity in the TAC, i.e. $\frac{{\rm{d}}{{\rm{A}}}_{{\rm{Notch}}}}{{\rm{dt}}}$,

8 $$\frac{{\rm{d}}{{\rm{A}}}_{{\rm{Notch}}}}{{\rm{dt}}}={{\rm{k}}}_{{\rm{Notch}}}\left(\frac{{{{\rm{A}}}_{{\rm{WntSC}}}}^{{\rm{n}}}}{1+{{{\rm{A}}}_{{\rm{WntSC}}}}^{{\rm{n}}}+{{{\rm{A}}}_{{\rm{YAP}}}}^{{\rm{n}}}{{+{\rm{A}}}_{{\rm{WntTA}}}}^{{\rm{n}}}}\right)-{{\rm{k}}}_{{\rm{dNotch}}}{{\rm{A}}}_{{\rm{Notch}}}+{{\rm{k}}}_{{\rm{PNotch}}}$$

where, ${{\rm{k}}}_{{\rm{dNotch}}}{{\rm{A}}}_{{\rm{Notch}}}$ is the first-order degradation rate in Notch activity and ${{\rm{k}}}_{{\rm{PNotch}}}$ is the generation of the Notch activity in the cell.

### Models of the Wnt activities in the SC and TAC

Since the Wnt activity in the SC is positively regulated by the Notch activity in the TAC (Fig. 8B, D), the rate of change in the Wnt activity, ${{\rm{A}}}_{{\rm{WntSC}}}$, in the SC, $\frac{{\rm{d}}{{\rm{A}}}_{{\rm{WntSC}}}}{{\rm{dt}}}$, is given as

9 $$\frac{{\rm{d}}{{\rm{A}}}_{\mathrm{WntSC}}}{\mathrm{dt}}={{\rm{k}}}_{\mathrm{WntSC}}\left(\frac{{{{\rm{A}}}_{\mathrm{Notch}}}^{{\rm{n}}}}{1+{{{\rm{A}}}_{\mathrm{Notch}}}^{{\rm{n}}}}\right)-{{\rm{k}}}_{\mathrm{dWntSC}}\,{{\rm{A}}}_{\mathrm{WntSC}}+{{\rm{k}}}_{\mathrm{PWntSC}}$$

where, k_WntSC_, k_dWntSC_, and k_PWntSC_ are parameters. ${{\rm{k}}}_{{\rm{dWntSC}}}{{\rm{A}}}_{{\rm{WntSC}}}$ is the degradation term and ${{\rm{k}}}_{{\rm{PWntSC}}}$ is the generation term. The generation of Wnt activity is assumed to be a constant.

On the other hand, the Wnt activity is suppressed by the Notch activity in the same cell (Fig. 8D). Thus, the rate of change in the Wnt activity ${{\rm{A}}}_{{\rm{WntTA}}}$ in the TAC, $\frac{{\rm{d}}{{\rm{A}}}_{{\rm{WntTA}}}}{{\rm{dt}}}$, is given as

10 $$\frac{{\rm{d}}{{\rm{A}}}_{{\rm{WntTA}}}}{{\rm{dt}}}={{\rm{k}}}_{{\rm{WntTA}}}\left(\frac{1}{1+{{{\rm{A}}}_{{\rm{Notch}}}}^{{\rm{n}}}}\right)-{{\rm{k}}}_{{\rm{dWntTA}}}{{\rm{A}}}_{{\rm{WntTA}}}+{{\rm{k}}}_{{\rm{PWntTA}}}$$

where, k_WntTA_, k_dWntTA_, and k_PWntTA_ are parameters.

### Model of the nuclear YAP activity in the TAC

Since nuclear YAP activity, A_YAP_, is positively regulated by the Notch activity (Fig. 8A, D), the rate of change in the nuclear YAP activity, $\frac{{\rm{d}}{{\rm{A}}}_{{\rm{YAP}}}}{{\rm{dt}}}$, in the TAC is given as:

11 $$\frac{{\rm{d}}{{\rm{A}}}_{\mathrm{YAP}}}{\mathrm{dt}}={{\rm{k}}}_{\mathrm{YAP}}\left(\frac{{{{\rm{A}}}_{\mathrm{Notch}}}^{{\rm{n}}}}{1+{{{\rm{A}}}_{\mathrm{Notch}}}^{{\rm{n}}}}\right)-{{\rm{k}}}_{\mathrm{dYAP}}\,{{\rm{A}}}_{\mathrm{YAP}}+{{\rm{k}}}_{\mathrm{PYAP}}$$

where, k_YAP_, k_dYAP_, and k_PYAP_ are parameters.

### Model of stochastic perturbations affecting Notch, Wnt, and Nuclear YAP activities in the TAC and Wnt activity in the SC

The rate of change in the signaling activities is given by ordinary differential equations (Eqs. (8)–(11)), comprising the regulatory, degradation, and generation terms. We extended these deterministic rate equations by introducing partial stochasticity in the model using Euler-Maruyama method^49^. The general form of the equation is given as:

$${{A}_{S}}_{i}\left(t+\Delta t\right)={{A}_{S}}_{i}\left(t\right)+{f}_{i}\left({A}_{S},t\right)\Delta t+\sigma \sqrt{\Delta t}\eta$$

where, ${{A}_{S}}_{i}$ is the stochastic signaling activity and ${f}_{i}({A}_{S},t)$ is the deterministic rate of change of the activity, given by Eqs. (8)–(11). The term $\sigma \sqrt{\Delta t}\eta$ is a continuous stochastic fluctuation in the activity added at each time step $\Delta t$, where, $\sigma$ is a parameter for the amplitude of random perturbations, and $\eta$ is a standard normally distributed random variable.

In the partial stochastic simulation, there are two perturbation terms: (1) Perturbations in the initial conditions, (2) Continuous random perturbations in the activities. Perturbations in the initial activity levels of Notch, Wnt, and nuclear YAP activities in the TACs, and Wnt activity in the SC reflect the biological variability in the initial states, and the small random fluctuations added continuously at each time step mimic random biological or environmental variabilities during the signaling process. This approach allows for investigating the robustness of the Notch-Wnt-YAP regulatory network in the TAC and SC under partial stochastic conditions.

### Materials

Dynamics of Notch, Wnt, and nuclear YAP activities in the TAC and Wnt activity in the stem cells as a function of time have been modeled using MATLAB R2023a. Equations (8–11) constitute a set of ODEs, which were solved numerically using the ode45 solver of MATLAB.

Further, for bifurcation analysis, Eqs. (8)–(11) were implemented in MATCONT, and parameter sweeps were performed. After specifying the model in MATCONT, the continuation of equilibria in one parameter was performed, and Hopf points were determined. Then, starting from a Hopf point, the continuation of the limit cycle was performed. Furthermore, Eqs. (8)–(11) were extended to include stochastic perturbation, resulting in stochastic differential equations, which were numerically integrated using the explicit Euler-Maruyama method with a fixed time step in MATLAB R2023a.

## Data Availability

All data supporting the findings of this study are available within the paper. Parameters used in this study can be found in this ref. ^50^. The data generated by the model can be obtained through the codes in the following link: https://github.com/raghvendra-singhIITK/epidermis.

## Code availability

The codes used in this study are available at: https://github.com/raghvendra-singhIITK/epidermis.
